# Genomic and evolutionary comparisons of diazotrophic and pathogenic bacteria of the order Rhizobiales

**DOI:** 10.1186/1471-2180-10-37

**Published:** 2010-02-08

**Authors:** Fabíola M Carvalho, Rangel C Souza, Fernando G Barcellos, Mariangela Hungria, Ana Tereza R Vasconcelos

**Affiliations:** 1 Laboratório Nacional de Computação Científica, Laboratório de Bioinformática, Av Getúlio Vargas 333, 25651-075, Petrópolis, Rio de Janeiro, Brazil; 2 Instituto Nacional de Metrologia, INMETRO, Av Nossa Senhora das Graças 50 - prédio 6, 25250-020, Xerém - Duque de Caxias, Rio de Janeiro - Brazil; 3 Embrapa Soja, Cx Postal 231, 86001-970 Londrina, Paraná, Brazil

## Abstract

**Background:**

Species belonging to the Rhizobiales are intriguing and extensively researched for including both bacteria with the ability to fix nitrogen when in symbiosis with leguminous plants and pathogenic bacteria to animals and plants. Similarities between the strategies adopted by pathogenic and symbiotic Rhizobiales have been described, as well as high variability related to events of horizontal gene transfer. Although it is well known that chromosomal rearrangements, mutations and horizontal gene transfer influence the dynamics of bacterial genomes, in Rhizobiales, the scenario that determine pathogenic or symbiotic lifestyle are not clear and there are very few studies of comparative genomic between these classes of prokaryotic microorganisms trying to delineate the evolutionary characterization of symbiosis and pathogenesis.

**Results:**

Non-symbiotic nitrogen-fixing bacteria and bacteria involved in bioremediation closer to symbionts and pathogens in study may assist in the origin and ancestry genes and the gene flow occurring in Rhizobiales. The genomic comparisons of 19 species of Rhizobiales, including nitrogen-fixing, bioremediators and pathogens resulted in 33 common clusters to biological nitrogen fixation and pathogenesis, 15 clusters exclusive to all nitrogen-fixing bacteria and bacteria involved in bioremediation, 13 clusters found in only some nitrogen-fixing and bioremediation bacteria, 01 cluster exclusive to some symbionts, and 01 cluster found only in some pathogens analyzed. In BBH performed to all strains studied, 77 common genes were obtained, 17 of which were related to biological nitrogen fixation and pathogenesis. Phylogenetic reconstructions for Fix, Nif, Nod, Vir, and Trb showed possible horizontal gene transfer events, grouping species of different phenotypes.

**Conclusions:**

The presence of symbiotic and virulence genes in both pathogens and symbionts does not seem to be the only determinant factor for lifestyle evolution in these microorganisms, although they may act in common stages of host infection. The phylogenetic analysis for many distinct operons involved in these processes emphasizes the relevance of horizontal gene transfer events in the symbiotic and pathogenic similarity.

## Background

The order Rhizobiales of alpha-Proteobacteria includes a variety of bacteria strategically important for their diversity in function and in niche occupancy. Studies of this order are thus interesting because it includes bacteria capable of fixing nitrogen when in symbiosis with leguminous plants, as well as obligate and facultative intracellular bacteria and animal and plant pathogens. Interestingly, these species with contrasting functionality share both some degree of genomic conservation and similarity among the symbiosis and pathogenicity strategies [1-4]; furthermore, these microorganisms take advantage of a variety of strategies to adapt and exploit ecological niches [5]. Altogether, genomic comparisons among symbiotic and pathogenic bacteria of the order Rhizobiales may provide significant insights about genetic variability, genome functionality, and operon organization of related species.

The nitrogen fixation ability in a free-living state is considered an ancient process; however, the evolution of the symbiosis with legumes was only possible due to the functional integration of the nodulation and nitrogen fixation genes over time. The ability to fix nitrogen has a more promiscuous nature, as observed in phylogenetic reconstructions of structural genes, such as the 16S rRNA, and *nif *and *fix *genes, while nodulation has a very specialized character which evolved in function of the host plant [6,7]. Finally, although nitrogen fixation and nodulation genes originated in divergent times, it is believed that through the mechanisms of gene transfer the genes related to both processes were grouped in operons and probably co-evolved in symbiotic bacteria [8]. Despite being widely distributed in the Archae and especially in the Bacteria domains, the process of biological nitrogen fixation is not monophyletic, with its origin and distribution being modified in function of selective pressures and processes as gene duplication, loss, and gene transfer [9-12].

Important not only as a factor contributing to the flexibility and adaptability of the genomes, the horizontal gene transfer is also involved in the generation of new symbiotic strains and might be the main factor responsible for the phylogenetic proximity between rhizobia and pathogenic bacteria, as well as for the similarities and/or divergences between the symbiosis and pathogenesis processes [13-19]. Transference may thus be the main factor explaining the presence of virulence genes in diazotrophic symbionts (e.g., homologous *virB1*-*virB11 *in *Rhizobium *(= *Agrobacterium*)*tumefaciens *and *Mesorhizobium loti *R7A) [20,21] as well as nitrogen-fixing genes in pathogenic bacteria (e.g. homologous to the cluster *fixNOQPGHIS *in the pathogens *Brucella melitensis *and *Pseudomonas aeruginosa*) [22]. In addition, it has also been demonstrated that the plant pathogen *R. tumefaciens *is capable of nodulating legumes after receiving a symbiotic plasmid [23]. However, until now, the functional evidence of the natural coexistence of genes for symbiosis and pathogenicity has been demonstrated only in strains of *R. rhizogenes *[24].

Despite the intriguing evolutionary questions raised in the analysis of symbiotic and pathogenic bacteria of the order Rhizobiales, very few studies of comparative genomics with a significant number of distinct genera and representative species of both lifestyles have been conducted between species of this prokaryotic order. In this study we have done such comparisons aiming at increasing the existent knowledge about the evolutionary divergence of these biological processes.

## Results

Phylogenetic reconstructions were performed in order to analyze the dynamics of the symbiosis and/or pathogenesis processes along the evolution of the species in study.

**Figure 1:**
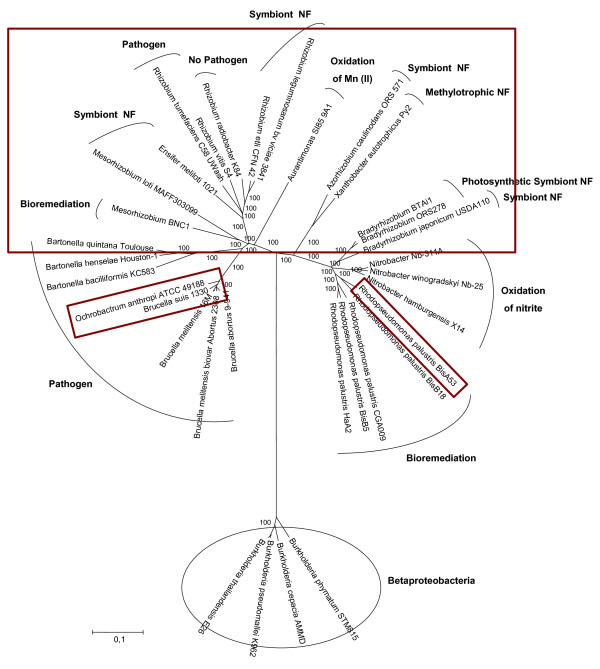
**Phylogeny model reconstructed with 104 housekeeping concatenated proteins of representatives of the Rhizobiales order**. Phylogeny model reconstructed with 104 housekeeping concatenated proteins of 30 strains (belonging to 25 species) of the order Rhizobiales. The Neighbor-Joining method was applied with Phylip 3.67 program and 1,000 replicates for bootstrap support. Representatives of the beta-Proteobacteria class were used as the outgroup. The 19 strains selected for the comparative analyses in this study are highlighted (Rhizobium sp. NGR234 is not included in this tree because its complete genome is not available).

The phylogenetic reconstruction model obtained with the 104 concatenated housekeeping proteins of 25 species and 30 strains with complete genome available presented a branched topology of two groups - one composed mostly of photosynthetic, methylotrophic, and bioremediation bacteria; and the second composed mostly of symbiotic and pathogenic bacteria. The second group is further subdivided into two major subgroups, one with the symbionts (except for *R. tumefaciens*, a pathogen showing high similarity with the symbionts), and another gathering the pathogens (Figure 1). Non-symbiotic nitrogen-fixing bacteria and bacteria involved in bioremediation closer to symbionts and pathogens in study may assist in the origin and ancestry genes and the gene flow occurring in Rhizobiales, and were considered in the comparisons.

**Figure 2:**
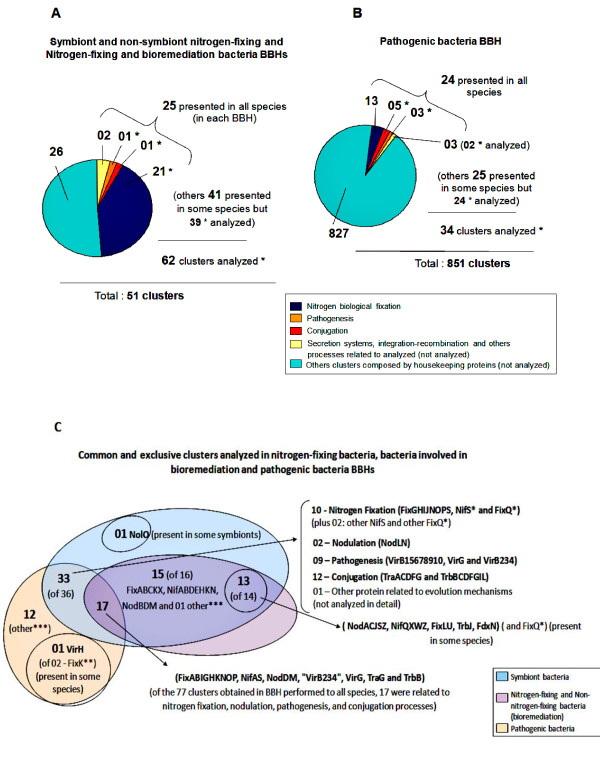
**Representation of the clusters obtained in BBH for biological nitrogen fixation, bioremediation, and pathogenesis processes**. Representation of the clusters obtained in BBH for each biological process. (A) BBH between symbiotic and non-symbiotic nitrogen-fixing bacteria and between nitrogen-fixing and bioremediation bacteria; (B) BBH between pathogenic bacteria; (C) the common and exclusives clusters analyzed in nitrogen-fixing bacteria, bacteria involved in bioremediation and pathogenic bacteria BBHs. (A)(B): * number of the clusters analyzed, total 96 clusters. (C): * repeat clusters obtained for NifS and FixQ. They are considered as unique NifS and unique FixQ in the analysis. (C): ** FixK was also identified in the BBH between nitrogen-fixing bacteria, but this cluster was not considered common for the bacterial analyzed because the cluster contained only one FixK present in *R. tumefaciens*. However, this protein was included in the FixK nitrogen-fixing cluster in phylogeny and presence and absence genes table. (C): *** Other clusters related to evolution mechanisms (not analyzed in detail).

From the 25 species used in the phylogenetic reconstruction, 19 were selected for comparative analysis (Additional file 1); in addition to *Rhizobium *sp. strain NGR 234. Four main Bidirectional Best Hits (BBH) were performed with the following genomic comparisons: i) symbiotic and non-symbiotic nitrogen-fixing bacteria; ii) nitrogen-fixing and bacteria involved in bioremediation; iii) pathogenic bacteria; and iv) considering all 19 species analyzed. In addition, two BBHs with lower stringency were performed, one for nitrogen-fixing bacteria and bacteria involved in bioremediation and another for pathogens, in order to identify clusters not obtained in the BBHs previously mentioned. To determine the common set of genes related to biological nitrogen fixation, a BBH was performed including genomic and plasmid sequences of symbiotic nitrogen-fixing bacteria and the non-symbiotic *Xanthobacter autotrophicus *Py2, and resulted in 51 clusters (Figure 2A). Considering the processes defined in the literature by using the model bacterium for symbiosis, *Bradyrhizobium japonicum *USDA 110 [25,26], of the 51 clusters identified, 23 are specific of biological nitrogen fixation, pathogenesis, and conjugation processes (Table A2a of supplementary material in database), in addition to 02 clusters related to protein secretion and integration and recombination processes (not analyzed) (Figure 2A).

Given the phylogenetic proximity observed in the reconstruction model between bacteria involved in bioremediation (*Rodopseudomonas palustris *BisA53), degradation of hydrocarbons (*Mesorhizobium *BNC1 and *X. autotrophicus *Py2) and oxidation of manganese (*Aurantimonas *SI85 9A1) with the symbiotic bacteria considered in this study, a BBH was performed to identify common genes to the processes of nitrogen fixation and bioremediation, and interestingly, the same clusters identified in the BBH of nitrogen-fixing bacteria were obtained in the BBH of nitrogen fixation and bioremediation (Table A2a of supplementary material in database).

In the BBH performed to identify common genes exclusive of pathogenic bacteria, 851 clusters were obtained (Figure 2B). From these, 24 clusters involved in pathogenicity, protein secretion, and integration-recombination processes were selected, based on the best studied plant pathogen, *Rhizobium tumefaciens *C58 [27-29] in addition to clusters involved in biological nitrogen fixation. *R. tumefaciens *was considered as the reference organism for pathogenesis because the symbionts in this study interact with plants and because in the animal pathogens of the Rhizobiales order, virulence-associated type IV secretion proteins homologous to *R. tumefaciens *were identified [30-32]. Of the 24 clusters obtained, 11 of these clusters were analyzed in this study. The remaining 13 are related to protein secretion and integration-recombination (Figure 2B) (Table A2b of supplementary material in database).

In the BBH performed with lower stringency for nitrogen-fixing bacteria and bacteria involved in bioremediation, 41 extra clusters of interest were selected (Figure 2A, and Table A2a of supplementary material in database); however, they did not include all bacteria used in the comparison. Among these clusters, two clusters were related to FixQ protein and two to NifS. Both FixQ and NifS clusters were composed by a separate group of bacteria. However, for each of these proteins, the clusters obtained were grouped in the analysis. Of the 41 clusters, 39 were analyzed. For pathogenic bacteria, of the clusters obtained in the analysis with lower stringency, 25 were obtained and 24 were selected for analysis (Figure 2B, and additional file 2) (in addition Table A2 of supplementary material in database).

In the BBHs performed in this study, except for clusters related to protein secretion and integration-recombination, 96 clusters were selected. Of these, 81 are common or exclusive to nitrogen-fixing bacteria, bacteria involved in bioremediation, and pathogenic bacteria BBHs. Of these, 63 were of interest for analysis (except the clusters related to other evolutive mechanisms and those repeats for the same protein, which were considered as one) (Figure 2). Among these 63 clusters, 33 were common to the process of biological nitrogen fixation and pathogenesis (10 of nitrogen fixation, 02 of nodulation, 09 of virulence, and 12 of conjugation), 15 clusters (NifABDEHKN, FixABCKX and NodBDM) were exclusive to all nitrogen-fixing bacteria and bacteria involved in bioremediation, 13 clusters (NodACJSZ, NifQXWZ, FixLU, TrbJ and FdxN) were found in only some nitrogen-fixing and bioremediation bacteria, 01 cluster (NolO) was exclusive to some symbionts, and 01 cluster (VirH) was found only in some pathogens (Figure 2C). The clusters common and unique to the groups mentioned above are presented in additional file 3. In the BBH performed to all strains studied, 77 common genes were obtained, of which 17 (FixA, FixB, FixI, FixG, FixH, FixK, FixN, FixO, FixP, NifA, NifS, NodD, NodM, "VirB234", VirG, TraG and TrbB) are related to biological nitrogen fixation and pathogenesis processes (Figure 2C).

**Figure 3:**
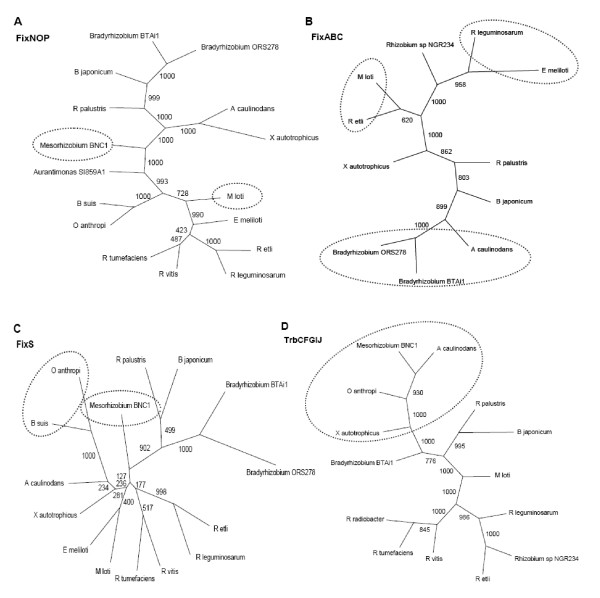
**FixNOP, FixABC, TrbCFGIJ and FixS phylogenies**. Phylogenies of selected nitrogen fixation and conjugation proteins obtained by BBH, reconstructed with the Neighbor-Joining method of the Phylip 3.67 program, with 1,000 replicates for bootstrap support. (A) concatenated phylogeny for FixNOP proteins; (B) concatenated phylogeny for FixABC proteins; (C) phylogeny for FixS protein; (D) concatenated phylogeny for TrbCFGIJ proteins.

**Figure 4:**
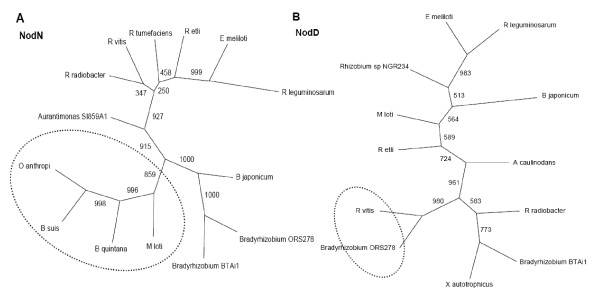
**NodN and NodD phylogenies**. Phylogenies of selected nodulation proteins obtained by BBH, reconstructed with the Neighbor-Joining method of the Phylip 3.67 program, with 1,000 replicates for bootstrap support. (A) phylogeny for NodN protein; (B) NodD protein.

**Figure 5:**
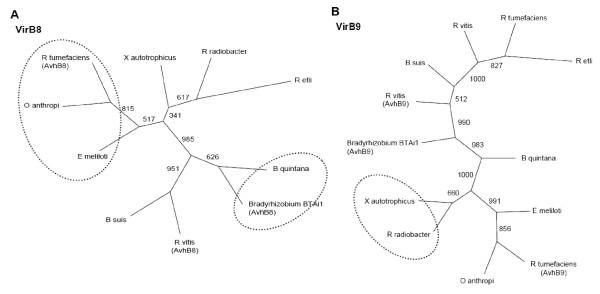
**VirB 8 and VirB9 phylogenies**. Phylogenies of selected proteins of type IV secretion system obtained by BBH reconstructed with the Neighbor-Joining method of the Phylip 3.67 program, with 1,000 replicates for bootstrap support. (A) phylogeny for VirB8 protein; (B) VirB9 protein.

Phylogenetic reconstructions were then performed to the proteins identified in the BBHs with more representativeness among the genomes analyzed. The topologies of Fix, Nif, Nod, Vir and Trb proteins (Figures 3 to 5, and additional file 4), have shown some incongruences when compared with the phylogeny model (Figure 1). The reconstruction obtained for FixNOP (Figure 3A) has a similar topology to the model with one exception. In the model reconstruction, *Mesorhizobium *BNC1 is close to the symbiont and pathogens branch, being grouped with *M. loti*, while in the FixNOP tree, *Mesorhizobium *BNC1 is distant from *M. loti*, in a highly reliable branch, suggesting that these genes in *Mesorhizobium *BNC1 could have originated from horizontal transfer.

The phylogenetic tree obtained with FixABC (Figure 3B) was the most distinct from the phylogeny model. In the group of photosynthetic, methylotrophic and bioremediation bacteria, *Azorhizobium caulinodans *is close to *Bradyrhizobium *and distant from *X. autotrophicus*. In the pathogen and symbiont group, *Rhizobium etli *is grouped with *M. loti *and not with *Rhizobium leguminosarum*, which in turn is grouped with *Ensifer *(= *Sinorhizobium*)*meliloti*, while in the phylogeny model this bacterium is more related to *M. loti*. Interestingly, the same patterns of FixABC were obtained in NifAB, with the grouping of *R. etli - M. loti *and *R. leguminosarum - E. meliloti *(additional file 4). Furthermore, the grouping between *R. etli *and *M. loti *and the proximity between *R. leguminosarum *and *E. meliloti *were also maintained in the reconstruction of NifDEKN, NifH, and NodABC (data not shown). In the reconstruction using FixH, *R. tumefaciens *appears to be more related to *E. meliloti *than with *Rhizobium vitis*, though with a low bootstrap support (additional file 4).

The FixS reconstruction (Figure 3C) is divergent from the model tree in respect to *Mesorhizobium *BNC1 and to the pathogens *Brucella suis *and *Ochrobactrum anthropi*. *Mesorhizobium *BNC1 was positioned in a separate branch and distant from *M. loti*, as also occurred in the reconstruction of FixNOP; in addition, *B. suis *and *O. anthropi *were closer to the nitrogen-fixing symbionts and methylotrophic bacteria. Although the grouping of *B. suis *and *O. anthropi *has high statistical support, inferences about the proximity of these pathogens with *A. caulinodans *and *X. autotrophicus *cannot be done because the internal nodes of the tree do not possess significant reliability values. A similar pattern to FixS was obtained with the TrbCFGIJ conjugation proteins (Figure 3D). *Mesorhizobium *BNC1 and the pathogen *O. anthropi *are closer to the symbiotic bacterium *A. caulinodans *and the methylotrophic bacterium *X. autotrophicus*, with high bootstrap support. In some of these species, transposases, integrases, and/or hypothetical proteins were identified next to TrbCFGIJ.

In relation to the nodulation genes, as to the model reconstruction (Figure 1), in the tree built with NodN, *M. loti *is close to the *O. anthropi*, *B. suis*, and *Bartonella quintana *pathogenic bacteria branch, with high reliability (Figure 4A).

The reconstruction with NodD (codified by *nodD *orthologous, preceded by *nodABC *genes) presented the most divergent topology among all trees obtained (Figure 4B). All groups are highly distinct from those observed in the model phylogeny, and then it was not possible to evidence the two main groups - one composed of photosynthetic, methylotrophic, and bioremediation bacteria, and another composed of symbiotic and pathogenic bacteria. Besides the discrepancy observed for the Nif and NodABC proteins between *R. etli - M. loti *and *R. leguminosarum - E. meliloti*, representatives of the genus *Rhizobium *(*Agrobacterium*) were more related to the genus *Bradyrhizobium *than among themselves. NodD and NodN were the only nodulation proteins found in the pathogen *R. vitis *and in the symbiont *Bradyrhizobium *ORS278, although this symbiont can nodulate without the involvement of *nod *genes [33]. In the NodD reconstruction, those species were grouped with high reliability.

The distinction between the two major groups - the first with symbionts and pathogens, and the second with photosynthetic, methylotrophic, and bioremediation bacteria - observed in the reconstruction model (Figure 1) was not evident in the VirB8, VirB9 (Figures 5A and 5B), and VirB10 phylogenies (additional file 4). In the topologies with these proteins, three patterns were maintained: i) *E. meliloti *was grouped with *R. tumefaciens *and *O. anthropi*; ii) *X. autotrophicus *had higher phylogenetic proximity with *Rhizobium radiobacter*; iii) and *Bradyrhizobium *BTAi1 was more related to *B. quintana *or *R. vitis*.

## Discussion

Despite the ecological and economical importance of the process of biological nitrogen fixation, and the intriguing evolutionary question about similarities and divergences in the symbiotic and pathogenic processes, there are very few studies of comparative genomics between these classes of prokaryotic microorganisms. The databank developed in this study offers an excellent opportunity for such studies, allowing the comparison of 30 strains of the order Rhizobiales with complete genomes available; in addition, the partial genome of the promiscuous strain NGR 234 of *Rhizobium sp*. was also included. The selected strains comprehend a good cover of the order Rhizobiales, including 26 species of 12 genera, classified in the main processes of biological nitrogen fixation, bioremediation, and pathogenesis. Certainly, the databank created in this study http://www.bnf.lncc.br/comparative will be useful for several future investigations, and in this study we have started by the comparison of the organisms using the approach of the Bidirectional Best Hits (BBH) method, selecting the proteins with higher similarity in sets of strains according to their function. From that, we built phylogenetic trees with different groups of concatenated proteins, to try to infer evolutionary pathways occurring in symbiotic and pathogenic Rhizobiales, focusing on genes known involved in these processes.

**Figure 6:**
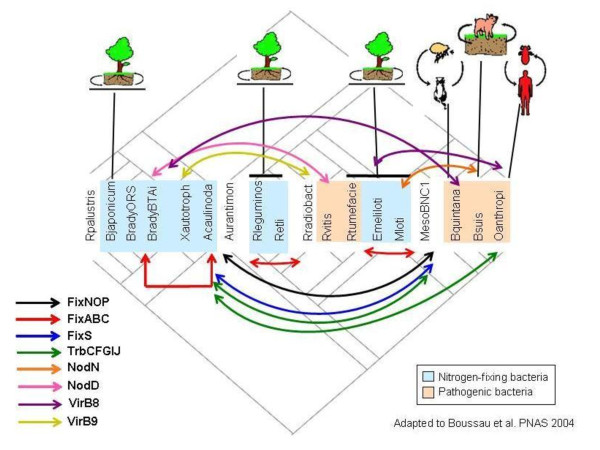
**Horizontal gene transfers in the evolution of Fix, Nod, Vir, and Trb proteins in Rhizobiales**. Model of the horizontal gene transfer events occurring to Fix, Nod, Vir, and Trb proteins in the Rhizobiales species studied.

**Figure 7:**
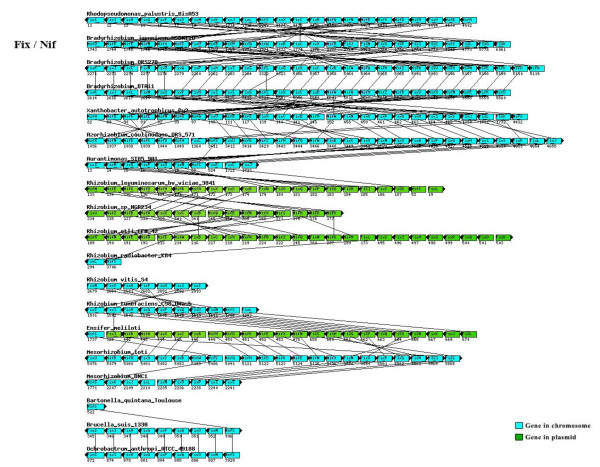
**Genomic location and the synteny to *fix-nif *genes of the Rhizobiales**. Genomic location and the synteny to *fix-nif *genes analyzed in the Rhizobiales species studied.

**Figure 8:**
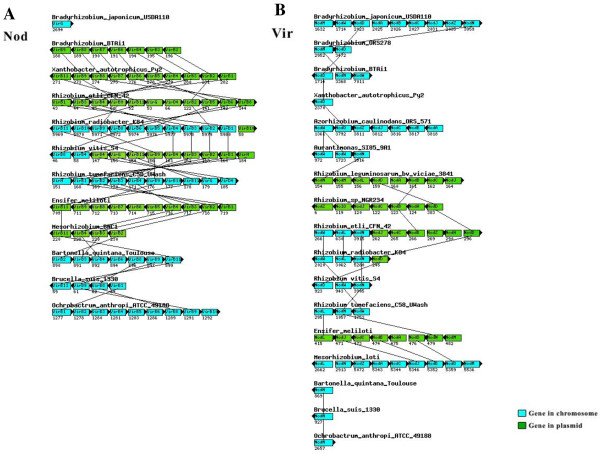
**Genomic location and the synteny to *nod*, and *vir *genes of the Rhizobiales**. Genomic location and the synteny to *nod *(A), and *vir *(B) genes analyzed in the Rhizobiales species studied.

**Figure 9:**
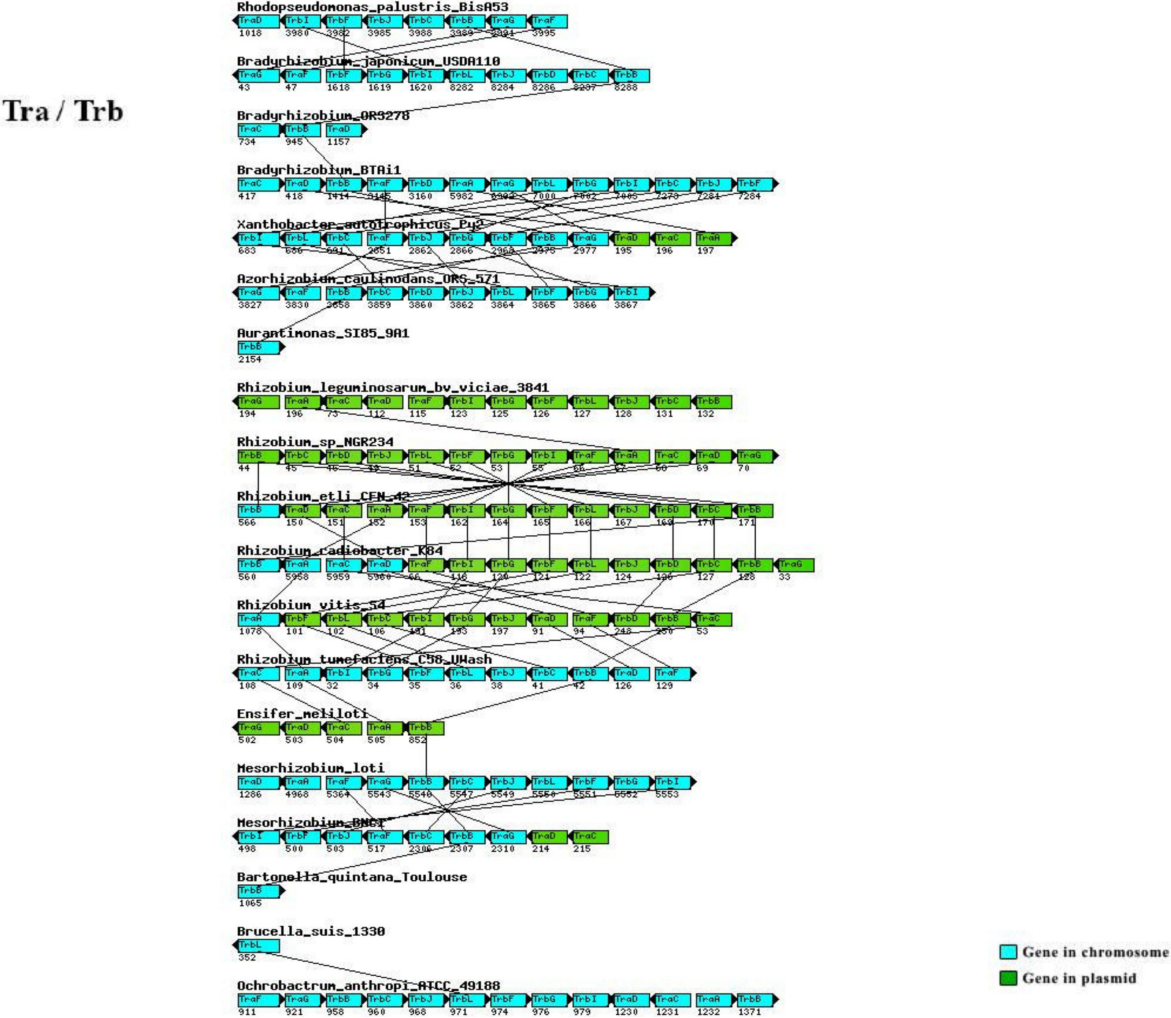
**Genomic location and the synteny to *tra- trb *genes of the Rhizobiales**. Genomic location and the synteny to *tra-trb *genes analyzed in the Rhizobiales species studied.

When compared with the phylogenetic model based on 104 housekeeping genes, divergence was observed in the Fix, Nif, Nod, Vir, and Trb topologies, and might be attributed to the high frequency of horizontal gene transfer (Figure 6), which has been reported in several of the representatives of the order Rhizobiales [34-39]. The genomic location and the synteny are important factors to be considered for horizontal gene transfer analysis in the genes analyzed. Many of the *fix*, *nif*, *nod*, *vir *and *trb *genes are located on plasmids or on chromosome in mobile elements called genomic islands. The disagreement observed in the reconstructions performed is corroborated by the absence of conservation of gene order to Fix, Nod, Vir, and Trb proteins (Figures 7 to 9).

Interesting data were observed especially in the comparison of symbiotic and pathogenetic bacteria. In the reconstruction using Fix proteins, the pathogenic and symbiotic species are more related to each other, except for FixABC. In this topology, the high reliability values associated with branches hint at least two possible moments of independent horizontal transfer events. In one moment, a horizontal transfer event would have occurred in *X. autotrophicus *and approximated this nitrogen-fixing methylotrophic bacteria to the non-photosynthetic symbiont group; and in another moment, two other independent events would have occurred between the nitrogen-fixing symbionts *R. etli - M. loti *and *R. leguminosarum - E. meliloti*.

In the topology built with the TrbCFGIJ proteins, a closer proximity between bioremediation bacteria, pathogenic, symbiotic, and non-symbiotic nitrogen-fixing bacteria was observed. TrbCFGIJ compose the *trb *operon, whose proteins form a membrane-associated macromolecular complex involved in mating-pair formation, facilitating the DNA transfer from donor to recipient cells [40]. The database built in this study shows that in the genomes of the bioremediatiors *Mesorhizobium *BNC1 and *R. palustris*, of the symbionts *A. caulinodans *and *B. japonicum *and of the methylotrophic nitrogen-fixing bacteria *X. autotrophicus*, there are transposases, integrases, and/or hypothetical proteins next to the TrbCFGIJ proteins, contrarily to the pathogenic *O. anthropi*. This observation suggests that these proteins may have been acquired through DNA transposition and/or integration mechanisms associated with horizontal gene transfer events, which occurred in the common ancestor of these species, and that other events of gene transfer may have occurred in *O. anthropi*, leading to its divergence from the other pathogens analyzed.

In NodN, as well as in FixH, FixNOP, VirB8, VirB9, and VirB10 topologies, the phylogenetic relationship observed between *M. loti *and the *Brucella-Bartonella *pathogens is corroborated by Paulsen et al. (2002) [3], which showed that *B. suis *presents high similarity to *R. tumefaciens*, *E. meliloti*, and *M. loti*, sharing extensive syntenic regions with the latter. Since NodN was the only nodulation protein present in all pathogens analyzed, in *R. radiobacter*, in photosynthetic nitrogen-fixing symbionts and other symbionts and in *Aurantimonas*, it is possible that this protein: i) has been acquired in an event preceding the separation between photosynthetic symbionts and pathogens, being lost in *A. caulinodans*, *X. autotrophicus*, and *Mesorhizobium *BNC1; or ii) that these organisms acquired this protein after the divergence between photosynthetic symbionts and pathogens, in a more recent horizontal transfer event. There is very little information about NodN. In *R. leguminosarum*, *nodN *is induced in response to flavonone molecules and this induction is *nodD*-dependent [41], and in both *R. leguminosarum *and *E. meliloti *it has been demonstrated that NodN is related to the root hair deformation phenotype, resulting from the action of Nod factors [42]. Therefore, although there are no experimental data on its biochemical function, in *E. meliloti *and *R. leguminosarum *it has been hypothesized that this protein may be involved in the synthesis and/or excretion of Nod factors [42]. In the pathogens analyzed in this study, NodN could have an auxiliary function during infection, modulating the induction of cell proliferation, since *Bradyrhizobium*, *Ensifer*, *Rhizobium*, *Brucella*, and *Bartonella *have similar strategies of infection, although the mechanisms are different [43].

The NodD reconstruction showed highly divergent; therefore, it was not possible to evidence the separation between photosynthetic, methylotrophic, and bioremediation bacteria from the group including symbiotic and pathogenic bacteria. The divergence observed might be related to NodD function in host-bacteria symbiosis. The host-bacteria specificity is established due to NodD-dependent upregulation of *nod *genes in response to flavonoids in the host plant's root exudates. NodD directly interacts with flavonoids to activate *nod *gene transcription, altering the response of the host cell according to the flavonoids secreted [44,45].

Although NodD is involved in activation of other nodulation genes, this protein belongs to the LysR-type transcriptional regulator family, which regulates a variety of genes, including those involved in virulence, quorum sensing, and motility [46]. Besides this, some species have more than one copy of the *nod*D gene. However, the phylogenetic analysis was performed using the peptide sequence codified by the *nodD *that precedes the operon *nodABC*. Since NodD can recognize different inducers, and the processes of infection and nodule formation require other determinants than these specific proteins, including other important proteins for the bacterium-host recognition [47], we may suppose that, in *R. vitis*, the *nodD *ortholog gene might be involved in the regulation of genes related to infection.

VirB8, VirB9, and VirB10 are transmembrane proteins that compose the type IV secretion system (T4SS), a structure consisting of several subunits that mediates the translocation of macromolecules by the cell envelope of Gram-positive and Gram-negative bacteria, used by many pathogens for the secretion of virulence determinants in the process of colonization of host tissues [48]. A type IV secretion system equivalent to that of plant pathogens has been described in the animal pathogens Bartonella and Brucella. In species of these genera, it has been demonstrated that VirB proteins are required in stages of the infection as colonization and inhibition of apoptosis and are essential for the virulence of some pathogens [49-51].

In the symbionts *E. meliloti *and *M. loti*, T4SS is not involved in the invasion and persistence of these microorganisms in their hosts [52]. In *E. meliloti *1021, this system is required for conjugation but not for symbiosis, whereas the analysis of *M. loti *R7A and MAFF303099 has shown that T4SS is involved in the symbiosis stabilization, increasing or decreasing the nodulation phenotype, according to the host involved [53].

The homologous proteins of *virB*, AvhB8, AvhB9, and AvhB10 genes identified in *R. tumefaciens *and VirB8, VirB9, and VirB10 of *E. meliloti *are located on plasmids. Although there is a considerable synteny between *R. tumefaciens *and *E. meliloti *chromosomes [5,26], conservation in the gene order among the plasmids of these microorganisms is not expected, due to the high frequency of horizontal gene transfer between plasmids of species of the Rhizobiales order. However, the grouping observed between the symbiont *E. meliloti *and the pathogen *R. tumefaciens *in the reconstruction trees generated with VirB8, VirB9, and VirB10 is in agreement with the topologies of VirB/Trb presented by Frank et al. (2005) [54], which examined the functional divergence and horizontal transfer of the T4SS. According to these authors, the coexistence of the AvhB conjugation protein with VirB translocation effectors in the same clade, as well as the location of these proteins in plasmids and the presence of multiple copies in some species, is indicative of the occurrence of multiple events of horizontal gene transfer, the process believed to be responsible for spreading the *virB *operon between the alpha-Proteobacteria, representing the dominant mechanism in the evolution of the conjugation systems for secretion. Regarding the proximity of the *X. autotrophicus *with *R. radiobacter*, and of *Bradyrhizobium *BTAi1 with *B. quintana *or *R. vitis*, there is no data in the literature that could allow inferences about such relationships. In these organisms, the *virB *operon is located between hypothetical and Tra conjugation proteins (data not shown). However, proteins involved in integration, transposition, and/or DNA recombination were not identified close to VirB8, VirB9, and VirB10 (database), which might allow inferences that these genes could have arisen from horizontal gene transfer.

## Conclusions

In this study, the genomic comparison has shown that symbiotic and pathogenic bacteria belonging to the order Rhizobiales may share several similar strategies of host interaction, inference taken from the high similarity on several proteins identified - e.g., FixNOPQ, NodN and VirB8910. However, it should be noted that some common clusters obtained are formed by protein families which may possess different functions in each process. The presence of symbiotic and virulence genes in both pathogens and symbionts does not seem to be the only determinant factor for lifestyle evolution in these microorganisms, although they may act in common stages of host infection. The phylogenetic analysis has also emphasized the relevance of horizontal gene transfer events to the evolution of the symbiotic and pathogenesis processes, contributing to genomic plasticity and evolution. There are no studies of comparative genomics in Rhizobiales with a focus on symbiosis and pathogenesis processes with the analyzed representative species of both lifestyles and showing phylogenetic analysis with many distinct operons involved in these processes. Besides this, the database offered by this study is the most representative for Rhizobiales until now and will also allow further important investigations that may help to infer crucial events that had contributed to the evolution of symbiosis of pathogenesis interactions.

## Methods

In order to select the species used for genomic comparison based on their phylogenetic proximity, a reconstruction with 30 bacteria belonging to the order Rhizobiales was obtained. The chosen strains belong to 25 different species and 12 genera and are shown in Figure 1. The reconstruction was performed by using a dataset consisting of 104 concatenated housekeeping proteins [55] based on the work of Williams *et al*. (2007) [56] and kindly provided by the authors, which showed a robust reconstruction for alpha-Proteobacteria. In addition to the species used by these authors, we included the sequences of *R. vitis *strain S4 and *R. radiobacter *strain K84, both previously classified in the genus *Agrobacterium *and both of whose genomes are available: strain S 4 is the pathogenic agent of crown gall disease in grapes, while strain K84 is non-pathogenic and has been developed for worldwide commercial use to control crown gall.

The tree generated was then established as the model phylogeny. From this tree, species with the largest phylogenetic proximity with the neighbor species of the other genera were selected, and representatives of the beta-Proteobacteria class were used as the outgroup.

Therefore, from the 30 species used in the reconstruction model (Figure 1), 19 were selected for comparative analysis (additional file 1). *Rhizobium sp*. NGR234 is not present in the reconstruction tree because some of the housekeeping proteins were not available, impairing the alignment. However, this bacterium was included in the comparison because it contains most of the genes analyzed in this study. *R. palustris *BisA53 was selected in preference to *Nitrobacter *Nb-31 1A because it is phylogenetically closely related to *B. japonicum*. *Mesorhizobium *BNC1 (an EDTA-degrading bacterium formerly known as *Agrobacterium *sp. BNC1), *Aurantimonas *SI85-9A1 (a marine bacterium known by its role in Mn(II) oxidation, and unusual in its feature of possessing both the large and small subunits of ribulose-1,5-bisphosphate carboxylase/oxygenase - RubisCO) and *X. autotrophicus *Py2 (a nitrogen-fixing methylotrophic, found in organic-rich soil, sediment, and water, and possessing genes responsible for alkene degradation) were selected by their proximity to the symbiotic bacteria in the phylogeny model (Figure 1), although they are not symbionts.

The genomic and/or plasmid sequences of the 19 bacterial species used in this study (Table A1) were acquired in the FTP format of the GenBank [57] and were used to obtain groups of ortholog genes (clusters) in genomes through the "Bidirectional Best Hits (BBH)" method [58]. This method compares the genome of each species against each other genome using the BLASTP (Basic Local Alignment Search Tool) program [59] to identify corresponding gene pairs recognized as the best hits in other genomes. BBHs among all functional groups (symbiotic, pathogenic and bioremediation-related), as well as between the species involved in each process, were performed using as parameters a coverage of 60% of the genome, 30% of identity, and e-value of 10^-5^.

For storage and analysis of data, a databank was developed in MySQL and Perl language [55]. The bank integrates tools and information from numerous biological databases as Interpro (The Integrated Resource of Protein Domains and Functional Sites) [60], Psort (Protein Subcellular Localization Prediction Tool) [61], KEGG (Kyoto Encyclopedia of Genes and Genomes) [62], COG (Clusters of Orthologous Groups of Proteins) [63], TCDB (Transporter Classification Database) [64], BlastP of KEGG and UniProt/Swiss-Prot [65], allowing several analyses as functional domains, subcellular localization, identification of metabolic pathways, genomic context, and alignment of proteins, among others. In addition, the databank allows automatic genomic comparisons by BBH between 31 species selected for study (the 30 bacteria shown in Figure 1 plus Rhizobium sp. NGR234) and the searches may be performed by gene name or synonym, sequence, and gene product. As the BBH method restricts the data to all selected species and as a gene may not be present in some species, comparisons with low stringency can be made applying an arbitrary minimum value of species compared within the interest set, making it possible to obtain more information. The databank is available at http://www.bnf.lncc.br/comparative.

For phylogenetic reconstructions, this study used the Neighbor-Joining method [66] of the Phylip (PHYLogeny Inference Package) [67] version 3.67 program [68], with resampling of 1000 replicates. Concatenated reconstructions were generated for proteins corresponding to genes organized in operons and identified in the same sample set. Unrooted reconstructions were generated for Fix, Nif, Nod, Vir, and Trb proteins, since it was not possible to use the same outgroup strains.

## Abbreviations

BBH: Bidirectional Best Hits method; T4SS: Type IV Secretion System; BLASTP: Basic Local Alignment Search Tool (for protein); Interpro: The Integrated Resource of Protein Domains and Functional Sites; Psort: Protein Subcellular Localization Prediction Tool; KEGG: Kyoto Encyclopedia of Genes and Genomes; COG: Clusters of Orthologous Groups of Proteins; TCDB: Transporter Classification Database; Phylip: Phylogeny Inference Package.

## Authors' contributions

FMC carried out the comparative genomic and evolution studies, the interpretation of data and the manuscript development. RCS developed the database and automated some data. FGB and MH have made substantial contributions to interpretation of data and have been involved in drafting the manuscript. ATRV conceived of the study and participated in coordination. All authors read and approved the final manuscript.

## Supplementary Material

Additional file 1 **Table A1.** Characteristics of the genomes of 19 Rhizobiales species compared in this study. Table showing the characteristics of the genomes of 19 Rhizobiales species compared in this study, as NCBI accession, genome length, number of plasmids, CG percent and host for each species. Click here for file

Additional file 2 **Table A2. **The number of clusters obtained in each comparative genomic performed by BBH. Table summarizing number of clusters obtained and analyzed in each comparative genomic performed by BBH. Click here for file

Additional file 3 **Tables A3 to 7**. Common and exclusive clusters analyzed in nitrogen-fixing bacteria, bacteria involved in bioremediation, and pathogenic bacteria BBHs presented by Fix, Nif, Nod, Vir, and Trb proteins. Table showing the presence and absence of the Fix, Nif, Nod, Vir, and Trb proteins analyzed in the clusters obtained in nitrogen-fixing bacteria, bacteria involved in bioremediation, and pathogenic bacteria BBHs. Click here for file

Additional file 4 **Figure S1.** NifAB, FixH, and VirB10 phylogenies. Phylogenies of selected clusters obtained by BBH, reconstructed with the Neighbor-Joining method of the Phylip 3.67 program, with 1,000 replicates for bootstrap support. (A) concatenated phylogeny for NifAB proteins; (B) phylogeny for FixH protein; (C) phylogeny for VirB10 protein. Click here for file
