# Supplementary figures and images for: Genomic and evolutionary comparisons of diazotrophic and pathogenic bacteria of the order Rhizobiales
Source: BMC Microbiol. 2010 Feb 8;10:37. doi: 10.1186/1471-2180-10-37 (PMC2907836; doi:10.1186/1471-2180-10-37)

##
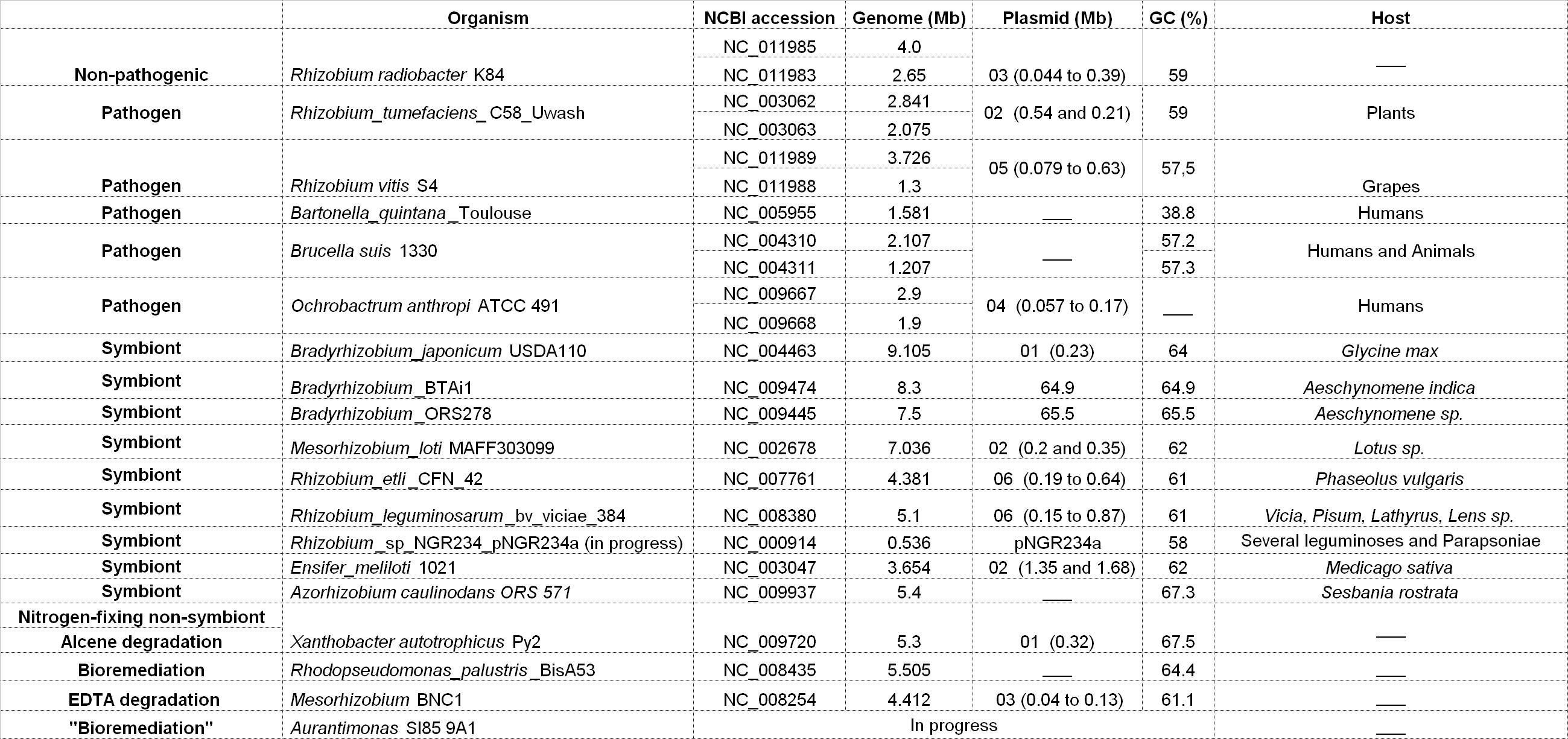

Supplement: Additional file 1 — Table A1. Characteristics of the genomes of 19 Rhizobiales species compared in this study. Table showing the characteristics of the genomes of 19 Rhizobiales species compared in this study, as NCBI accession, genome length, number of plasmids, CG percent and host for each species. [file 1471-2180-10-37-S1.DOC]
