# Supplementary material for: Genomic and evolutionary comparisons of diazotrophic and pathogenic bacteria of the order Rhizobiales
Source: BMC Microbiol. 2010 Feb 8;10:37. doi: 10.1186/1471-2180-10-37 (PMC2907836; doi:10.1186/1471-2180-10-37)
Supplement: Additional file 2 — Table A2. The number of clusters obtained in each comparative genomic performed by BBH. Table summarizing number of clusters obtained and analyzed in each comparative genomic performed by BBH. [file 1471-2180-10-37-S2.DOC]

Table 2. The number of clusters obtained in each comparative genomic performed by BBH.

| Comparative genomic by Best Bidirectional Hit (BBH) | Processes | Number of clusters obtained |
| --- | --- | --- |
| Symbiont and non-symbiont nitrogen-fixing bacteria  and  Nitrogen-fixing and bacteria involved in bioremediation | Biological nitrogen fixation, pathogenesis, conjugation, secretion systems, and integration-recombination | 25 clusters common to all species compared |
|  | Cell maintenance | 26 clusters common to all species compared |
|  | Biological nitrogen fixation, pathogenesis, and conjugation | 23 clusters analyzed of 25 obtained |
|  |  | 41 clusters presented in some species compared |
|  |  | 39 clusters analyzed * of 41 obtained |
| * In BBH performed with lower stringency, two clusters were obtained to FixQ protein for some species. These clusters are composed by different nitrogen-fixing species. Both clusters were grouped in the analysis and considered as only one cluster for FixQ. | | |
| Pathogenic bacteria | Pathogenesis, conjugation biological nitrogen fixation, secretion systems, and integration-recombination | 24 clusters common to all species compared |
|  | Cell maintenance | 827 clusters presented in some species of all compared |
|  | Pathogenesis, conjugation, and biological nitrogen fixation | 11 clusters analyzed * of 24 obtained |
|  |  | 25 present in some species compared |
|  |  | 24 analyzed ** of 25 obtained present in some species compared |
| * Two clusters were obtained to NifS protein. These clusters were grouped in the analysis and considered as only one cluster for NifS.  ** In pathogen BBH was obtained the cluster “Transcricional regulator/ FixK”. Although FixK was also identified in the BBH between nitrogen-fixing bacteria, the cluster was not considered common for the bacterial analyzed because the cluster contained only one FixK, it present in *R. tumefaciens*. However, this protein was included in the FixK nitrogen-fixing cluster in phylogeny and presence and absence genes table. | | |
| Nitrogen-fixing (symbiont and non-symbiont) bacteria, bacteria involved in bioremediation and pathogenic bacteria | Pathogenesis, conjugation biological nitrogen fixation, secretion systems, integration-recombination, and cell maintained | 77 clusters common to all species compared |
|  | Pathogenesis, conjugation, and biological nitrogen fixation | 17clusters common to all species compared |
