# Supplementary material for: Genomic and evolutionary comparisons of diazotrophic and pathogenic bacteria of the order Rhizobiales
Source: BMC Microbiol. 2010 Feb 8;10:37. doi: 10.1186/1471-2180-10-37 (PMC2907836; doi:10.1186/1471-2180-10-37)
Supplement: Additional file 3 — Tables A3 to 7. Common and exclusive clusters analyzed in nitrogen-fixing bacteria, bacteria involved in bioremediation, and pathogenic bacteria BBHs presented by Fix, Nif, Nod, Vir, and Trb proteins. Table showing the presence and absence of the Fix, Nif, Nod, Vir, and Trb proteins analyzed in the clusters obtained in nitrogen-fixing bacteria, bacteria involved in bioremediation, and pathogenic bacteria BBHs. [file 1471-2180-10-37-S3.DOC]

Table 3. Common and exclusive clusters analyzed in nitrogen-fixing bacteria, bacteria involved in bioremediation and pathogenic bacteria BBHs presented by Fix proteins.

| Organism | | FixA / EtfB* | FixB / EtfA* | FixC | FixG | FixH | FixI | FixJ / NodW***** | FixK | FixL | FixN | O | P | Q | S | | U | | X |  |
| --- | --- | --- | --- | --- | --- | --- | --- | --- | --- | --- | --- | --- | --- | --- | --- | --- | --- | --- | --- | --- |
| Non-pathogen | Rhizobium radiobacter K84 | * | * |  |  |  |  | * |  | + |  |  |  |  | |  | |  |  | |
| Pathogen | Rhizobium tumefaciens C58 UWash | * | * |  | + | + | + | * | * | + | + | + | + | + | | + | |  |  | |
|  | Rhizobium vitis S4 | * | * |  |  | Avi2391 | + | * |  |  | + | + | + | + | | + | |  |  | |
|  | Bartonella quintana Toulouse | * | * |  |  |  |  |  |  |  |  |  |  |  | |  | |  |  | |
|  | Brucella suis 1330 | * | * |  | + | + | + |  |  |  | + | + | + | + | | + | |  |  | |
|  | Ochrobactrum anthropi ATCC 49188 | * | * |  | + | + | + |  |  |  | + | + | + | + | | + | |  |  | |
| Symbiont | Bradyrhizobium BTAi1 | + * | + * | + | + | + | + | + * | + | + | + | + | + |  | | + | | + | + | |
|  | Bradyrhizobium japonicum | + * | + * | + | + |  | + | + * | + | + | + | + | + |  | | + | | + | + | |
|  | Bradyrhizobium ORS278 | + * | + * | + | + | + | + | + | + | + | + | + | + | * | | + | | + | + | |
|  | Mesorhizobium loti | + | + | + | + | + | + | + |  |  | + | + | + |  | | + | | + | + | |
|  | Rhizobium etli CFN42 | + * | + * | + | + | + | + | * | + | + | + | + | + | + | | + | |  | + | |
|  | Rhizobium leguminosarum viciae 3841 | + | + | + | + | + | + | pRL120792* | + | p pRL90020 | + | + | + | + | | + | |  | + | |
|  | Rhizobium sp NGR234 (in progress) | + | + | + |  |  |  |  |  |  |  |  |  |  | |  | | + | + | |
|  | Ensifer meliloti | + * | + * | + | + | + | + | + | + | + | + | + | + | + | | + | |  | + | |
|  | Azorhizobium caulinodans ORS571 | + * | + * | + | + | + | + | + * | + | + | + | + | + | * | | + | | + | + | |
| Non-symbiont nitrogen-fixing | Xanthobacter autotrophicus Py2 | + * | + * | + | + | + | + | + | + | + | + | + | + |  | | + | | + | + | |
| EDTA degradation | Mesorhizobium BNC1 | * | * |  |  |  | + | + | + | + | + | + | + |  | | + | |  |  | |
| Biorremediation | Rhodopseudomonas palustris BisA53 | + * | + * | + | + | + | + | + | + | + | + | + | + |  | | + | | + | + | |
| “Biorremediation” | Aurantimonas SI85 9A1 (in progress) | * | * |  | + | + | + | + * | + | + | + | + | + |  | |  | |  |  | |

FixAEtfB and FixBEtfA: The clusters were formed by FixAB and EtfAB related proteins; * Etf were identified; + * - both Fix and Etf were identified; + - Fix were identified

FixK – * only FixK was identified in cluster obtained in pathogen BBH. In the analysis, this protein was included in FixK clusters of nitrogen-fixing bacteria

NodW/FixJ - The clusters were formed by NodW and FixJ proteins; * corresponds to NodW protein; + * - both NodW and FixJ were identified; + - FixJ were identified

FixQ – There are two clusters for FixQ composed by different groups of bacteria; * - FixQ presents in other cluster

The code proteins are the proteins obtained in Rhizobase Database, not present in the clusters obtained by BBH.

Table 4. Common and exclusive clusters analyzed in nitrogen-fixing bacteria, bacteria involved in bioremediation and pathogenic bacteria BBHs presented by Nif proteins.

| Organism | | NifA /  NtrCX* | NifB | NifD | NifE | NifH | NifK | NifN | NifQ | NifS | NifX | NifW | NifZ | FdxN |
| --- | --- | --- | --- | --- | --- | --- | --- | --- | --- | --- | --- | --- | --- | --- |
| Non-pathogen | Rhizobium radiobacter K84 | * |  |  |  |  |  |  |  | + |  |  |  |  |
| Pathogen | Rhizobium tumefaciens C58 UWash | * |  |  |  |  |  |  |  | + |  |  |  |  |
|  | Rhizobium vitis S4 | * |  |  |  |  |  |  |  | + |  |  |  |  |
|  | Bartonella quintana Toulouse | * |  |  |  |  |  |  |  | + |  |  |  |  |
|  | Brucella suis 1330 | * |  |  |  |  |  |  |  | + |  |  |  |  |
|  | Ochrobactrum anthropi ATCC 49188 | * |  |  |  |  |  |  |  | + |  |  |  |  |
| Symbiont | Bradyrhizobium BTAi1 | + * | + | + | + | + | + | + | + | + | + | + | + | + |
|  | Bradyrhizobium japonicum | + * | + | + | + | + | + | + | + | + | + | + |  | + |
|  | Bradyrhizobium ORS278 | + * | + | + | + | + | + | + | + | + | + | + | + | + |
|  | Mesorhizobium loti | + | + | + | + | + | + | + | + | + | + | + |  | + |
|  | Rhizobium etli CFN42 | + * | + | + | + | + | + | + | + | + | + | + | RESP0086R | + |
|  | Rhizobium leguminosarum viciae 3841 | + | + | + | + | + | + | + |  | p  pRL80059 | p  pRL100157 |  |  |  |
|  | Rhizobium sp NGR234 (in progress) | + | + | + | + | + | + | + | + | + | + | NGR243_432 | NGR234_396 | + |
|  | Ensifer meliloti | + * | + | + | + | + | + | + |  | + | + |  |  | + |
|  | Azorhizobium caulinodans ORS571 | + * | + | + | + | + | + | + | + | + | + | + | + | + |
| Non-symbiont nitrogen-fixing | Xanthobacter autotrophicus Py2 | + * | + | + | + | + | + | + | + | + | + | + | + | + |
| EDTA degradation | Mesorhizobium BNC1 | * |  |  |  |  |  |  |  | + |  |  |  |  |
| Bioremediation | Rhodopseudomonas palustris BisA53 | + * | + | + | + | + | + | + | + | + |  |  |  |  |
| “Bioremediation” | Aurantimonas SI85 9A1 (in progress) | * |  |  |  |  |  |  |  | + |  |  |  |  |

NifA/NtrCX: The clusters were formed by NifA and NtrC or NtrX related proteins; * corresponds to Ntr proteins; + * - both NifA and Ntr were identified; + - NifA were identified.

The code proteins are the proteins obtained in Rhizobase Database, not present in the clusters obtained by BBH.

Table 5. Common and exclusive clusters analyzed in nitrogen-fixing bacteria, bacteria involved in bioremediation and pathogenic bacteria BBHs presented by Nod proteins.

| Organism | | NodA | NodB | NodC | NodD* | NodJ | NodL | NodM* | NodN | NolO | NodS | NodZ |
| --- | --- | --- | --- | --- | --- | --- | --- | --- | --- | --- | --- | --- |
| Non-pathogen | Rhizobium radiobacter K84 |  |  |  | +* |  | + |  | + |  |  |  |
| Pathogen | Rhizobium tumefaciens C58 UWash |  |  |  |  |  | + |  | + |  |  |  |
|  | Rhizobium vitis S4 |  |  |  | +* |  |  |  | + |  |  |  |
|  | Bartonella quintana Toulouse |  |  |  |  |  |  |  | + |  |  |  |
|  | Brucella suis 1330 |  |  |  |  |  |  |  | + |  |  |  |
|  | Ochrobactrum anthropi ATCC 49188 |  |  |  |  |  |  |  | + |  |  |  |
| Simbiont | Bradyrhizobium BTAi1 |  |  |  | + |  |  |  | + |  |  |  |
|  | Bradyrhizobium japonicum | + | + | + | + | + |  | + | + | + | + | + |
|  | Bradyrhizobium ORS278 |  |  |  | + |  |  |  | + |  |  |  |
|  | Mesorhizobium loti | + | + | mlr6163 | + | + | mll2768 | mlr6386 | mlr3097 | + | + | + |
|  | Rhizobium etli CFN42 | + | + | + | + | + | + |  | + | + | + | + |
|  | Rhizobium leguminosarum viciae 3841 | + | + | + | + | + | + | + | + |  |  |  |
|  | Rhizobium sp NGR234 (in progress) | NGR234_138 | + | NGR234_136 | + | NGR234_134 |  |  |  | NGR234_133 | NGR234_251 | NGR234_008 |
|  | Ensifer meliloti | SMa0869 | + | + | + | + | + | + | + | + |  |  |
|  | Azorhizobium caulinodans ORS571 | + | + | AZC_3816 | + | + |  |  |  |  | + | + |
| Non-symbiont nitrogen-fixing | Xanthobacter autotrophicus Py2 |  |  |  | + |  |  |  |  |  |  |  |
| EDTA degradation | Mesorhizobium BNC1 |  |  |  |  |  |  |  |  |  |  |  |
| Biorremediation | Rhodopseudomonas palustris BisA53 |  |  |  |  |  |  |  |  |  |  |  |
| “Biorremediation” | Aurantimonas SI85 9A1 (in progress) |  |  |  |  |  |  |  | + |  |  |  |

* The clusters were common the nitrogen-fixing and pathogenic bacteria BBH but the proteins in pathogens are not correspond to NodDM.

+* NodD was identified by Blast. In BBH, this protein was not obtained.

The code proteins are the proteins obtained in Rhizobase Database, not present in the clusters obtained by BBH.

Table 6. Common and exclusive clusters analyzed in nitrogen-fixing bacteria, bacteria involved in bioremediation and pathogenic bacteria BBHs presented by Vir proteins.

| Organism | | Avh/VirB1 | Avh/VirB234 | VirB5 | VirB6 | VirB7 | VirB8 | VirB9 | VirB10 | VirG | VirH |
| --- | --- | --- | --- | --- | --- | --- | --- | --- | --- | --- | --- |
| Non-pathogen | Rhizobium radiobacter K84 | + | +++ | + | + |  | + | + |  |  |  |
| Pathogen | Rhizobium tumefaciens C58 UWash | + | +++ | + | + |  | + | + | + | + |  |
|  | Rhizobium vitis S4 | + | +++ |  |  |  | + | + | + | + |  |
|  | Bartonella quintana Toulouse |  | +++ |  | + |  | + | + |  |  |  |
|  | Brucella suis 1330 | + |  |  |  |  | + | + |  |  |  |
|  | Ochrobactrum anthropi ATCC 49188 | + | +++ | + | + |  | + | + |  |  |  |
| Symbiont | Bradyrhizobium BTAi1 |  | +++ |  | + | + | + | + | + |  |  |
|  | Bradyrhizobium japonicum |  |  |  |  |  |  |  |  | + |  |
|  | Bradyrhizobium ORS278 |  |  |  |  |  |  |  |  |  |  |
|  | Mesorhizobium loti |  |  |  |  |  |  |  |  |  |  |
|  | Rhizobium etli CFN42 | + | +++ | + | + |  | + | + | + | + |  |
|  | Rhizobium leguminosarum viciae 3841 |  |  |  |  |  |  |  |  |  |  |
|  | Rhizobium sp NGR234 (in progress) |  |  |  |  |  |  |  |  |  |  |
|  | Ensifer meliloti | + | +++ | + | + | + | + | + | + |  |  |
| FBN | Azorhizobium caulinodans ORS571 |  |  |  |  |  |  |  |  |  |  |
|  | Xanthobacter autotrophicus Py2 | + | +++ | + | + | + | + | + | + |  |  |
| Biorremediation | Mesorhizobium BNC1 |  | +++ |  |  |  |  |  |  |  |  |
|  | Rhodopseudomonas palustris BisA53 |  |  |  |  |  |  |  |  |  |  |
| Oxidation of Mn | Aurantimonas SI85 9A1 (in progress) |  |  |  |  |  |  |  |  |  |  |

+++ VirB2, VirB3 and VirB4 (or their Avh homologous) were identified.

Table 7. Common and exclusive clusters analyzed in nitrogen-fixing bacteria, bacteria involved in bioremediation and pathogenic bacteria BBHs presented by Tra/Trb proteins.

| Organism | | TraA | TraC | TraD | TraF | TraG  VirD4* | TrbB/CpaF CtpG/  VirB11* | TrbC | TrbD | TrbF | TrbG | TrbI | TrbJ | TrbL |
| --- | --- | --- | --- | --- | --- | --- | --- | --- | --- | --- | --- | --- | --- | --- |
| Non-pathogen | Rhizobium radiobacter K84 | Arad14094 | + | + | + | + | +++ | + | + | + | + | + | Arad14175 | + |
| Pathogen | Rhizobium tumefaciens C58 UWash | + | + | + | + | ++ | +++ | + |  | + | + | + | Atu6037 | + |
|  | Rhizobium vitis S4 | Avi0682 | + | + | + | ++ | +++ | + | + | + | + | + | Avi9263 | + |
|  | Bartonella quintana Toulouse |  |  |  |  |  | + * |  |  |  |  |  |  |  |
|  | Brucella suis 1330 |  |  |  |  |  | + * |  |  |  |  |  |  | + |
|  | Ochrobactrum anthropi ATCC 49188 | + | Oant_0649 | Oant_0648 | + | + | ++ | + |  | + | + | + | Oant_3216 | + |
| Symbiont | Bradyrhizobium BTAi1 | BBta_6372 | p BBta_0436 | + | + | p  BBta_p0247*  + | +++ | + | BBta_3366 | + | + | + | + | + |
|  | Bradyrhizobium japonicum |  |  |  | + | + | ++ | p bll8287 | p  bsl8286 | + | + | + | p  bll8284 | + |
|  | Bradyrhizobium ORS278 | BRADO0796 | p BRADO0797 | BRADO1240 |  | + | +c |  |  |  |  |  |  |  |
|  | Mesorhizobium loti | mll5729 |  | p msr0965 | + | + | + | + |  | + | + | + | + | + |
|  | Rhizobium etli CFN42 | RHE_CH00726 | + | + | + | + * | +++ | + | + | + | + | + | RHE_PA00174 | + |
|  | Rhizobium leguminosarum viciae 3841 | pRL100219 | pRL70085 | pRL80120A | pRL80123 | + | p pRL80142 | p pRL80141 |  | p pRL80136 | p  pRL80135 | p  pRL80133 | p pRL80138 | p  pRL80137 |
|  | Rhizobium sp NGR234 (in progress) | NGR234_072 | + | + | + | y4dV | + | + | + | + | + | + | NGR234_053 | + |
|  | Ensifer meliloti | SMa0934 | + | SMa0930 |  | SMa0929 | +c * |  |  |  |  |  |  |  |
| FBN | Azorhizobium caulinodans ORS571 |  |  |  | + | + | + | + | AZC_3860 | + | + | + | + | + |
|  | Xanthobacter autotrophicus Py2 | Xaut_5003 | + | + | + | + | + * | + |  | + | + | + | + | + |
| Biorremediation | Mesorhizobium BNC1 |  | + | + | + | + | +++ | + |  | + | + | + | + | + |
|  | Rhodopseudomonas palustris BisA53 |  |  | + | + | + | ++ | + |  | + | + | + | RPE_4020 | + |
| Oxidation of Mn | Aurantimonas SI85 9A1 (in progress) |  |  |  |  |  | + c |  |  |  |  |  |  |  |

TraG/VirD4 - + * both proteins are present

TrbB/ CpaF or CtpG/ VirB11 - + TrbB is presents; ++ TrbB and Cpa or CtpG are present; +++ TrbB, CpaF or CtpG and VirB11 are present

+ * TrbB and VirB11 were identified, +c Cpa or CtpG was identified, +c * Cpa or CtpG and VirB11 were identified.

The code proteins are the proteins obtained in Rhizobase Database, not present in the clusters obtained by BBH
