# Supplementary material for: Genomic and evolutionary comparisons of diazotrophic and pathogenic bacteria of the order Rhizobiales
Source: BMC Microbiol. 2010 Feb 8;10:37. doi: 10.1186/1471-2180-10-37 (PMC2907836; doi:10.1186/1471-2180-10-37)
Supplement: Supplementary file 4 [file 1471-2180-10-37-S4.DOC]

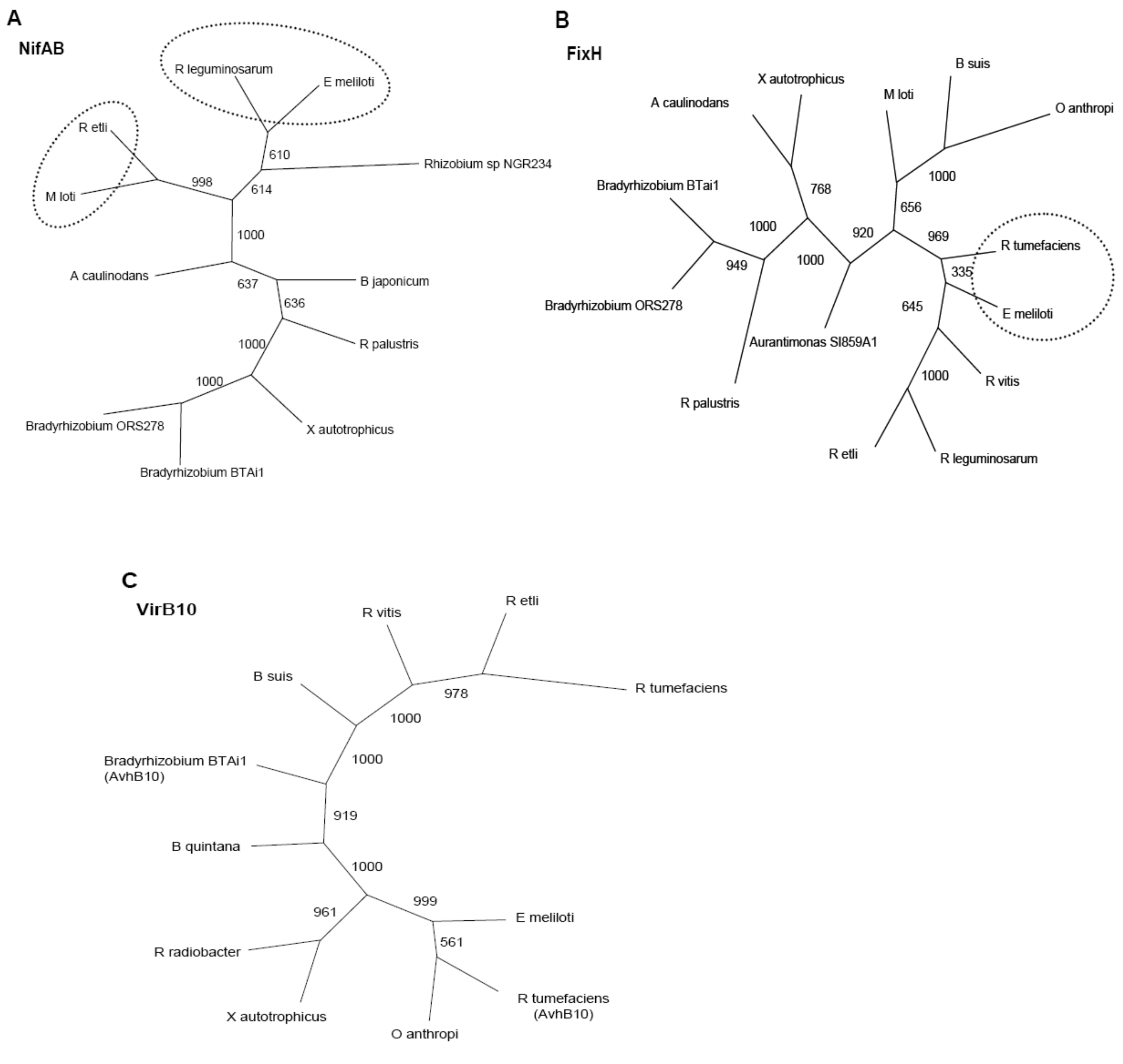
A
NifAB
R leguminosarum
E meliloti
R etli
M loti
998
610
614
Rhizobium sp NGR234
1000
A caulinodans
637
B japonicum
636
R palustris
1000
1000
Bradyrhizobium ORS278
X autotrophicus
Bradyrhizobium BTAi1
B
FixH
X autotrophicus
A caulinodans
M loti
B suis
O anthropi
Bradyrhizobium BTai1
768
1000
656
1000
920
969
R tumefaciens
949
1000
335
Bradyrhizobium ORS278
645
E meliloti
Aurantimonas SI859A1
1000
R vitis
R palustris
R etli
R leguminosarum
C
VirB10
R vitis
R etli
B suis
978
R tumefaciens
1000
Bradyrhizobium BTAi1
(AvhB10)
1000
919
B quintana
1000
961
999
E meliloti
R radiobacter
561
R tumefaciens
(AvhB10)
X autotrophicus
O anthropi
